# Internal gallbladder drainage prevents development of acute cholecystitis in a pig model: a randomized study

**DOI:** 10.1186/1750-1164-4-4

**Published:** 2010-05-26

**Authors:** Daniel W Kjaer, Frank V Mortensen, Jens K Møller, Stephen J Hamilton-Dutoit, Peter Funch-Jensen

**Affiliations:** 1 Surgical Gastroenterological Department L, Aarhus University Hospital, Aarhus, Denmark; 2 Department of Clinical Microbiology, Aarhus University Hospital, Skejby, Denmark; 3 Institute of Pathology, Aarhus University Hospital, Aarhus, Denmark

## Abstract

**Background:**

Acute cholecystitis can be the result of retention of bile in the gallbladder with possible secondary infection and ischaemia. The aim of the present study was to investigate whether internal drainage of the gallbladder could protect against the development of acute cholecystitis in a pig model.

**Materials and methods:**

Twenty pigs were randomized to either internal drainage (drained) or not (undrained). Day 0 acute cholecystitis was induced by ligation of the cystic artery and duct together with inoculation of bacteria. Four days later the pigs were killed and the gallbladders were removed and histologically scored for the presence of cholecystitis. Bile and blood samples were collected for bacterial culturing and biochemical analyses.

**Results:**

The histological examination demonstrated statistical significant differences in acute cholecystitis development between groups, the degree of inflammation being highest in undrained pigs. There were no differences in bacterial cultures between the two groups.

**Conclusion:**

Internal drainage of the gallbladder protected against the development of acute cholecystitis in the present pig model. These findings support the theory that gallstone impaction of the cystic duct plays a crucial role as a pathogenetic mechanism in the development of acute cholecystitis and suggest that internal drainage may be a way to prevent and treat acute cholecystitis.

## Introduction

Gallstones are common in patients throughout the western world and are found in about 10% of the adult population [1]. Gallstone related disease is one of the most frequent medical problems demanding surgical intervention. In Denmark, 130 per 100.000 inhabitants are cholecystectomized each year [2], most often because of biliary attacks of pain. The annual incidence of acute cholecystitis, the second largest group undergoing cholecystectomy, is approximately 20 per 100.000 inhabitants [2]. In a subgroup of patients with acute cholecystitis, surgical intervention is hazardous due to poor performance status. In these patients, the standard treatment options have traditionally been conservative treatment or percutaneous transperitoneal cholecystostomy (PTCS) [3-7]. A significant drawback with both these treatment modalities is a high rate of recurrences, reported to range from 15% to 47% [8,9]. In recent years, an alternative treatment option, endoscopic gallbladder drainage (EGBD), has emerged [10-14]. One theoretical advantage of EGBD compared with PTCS, is that treatment can be sustained for longer periods, because there is not, as in PTCS, an external component of the drain. This possibility for prolonged treatment raises the hope that EGBD may be a more definitive treatment of cholecystitis than PTCS.

Calculous cholecystitis is thought to develop when the cystic duct becomes obstructed by an impacting gallstone. Secondary bacterial contamination of the stagnant bile seems an obvious mechanism for exacerbation in the development of acute cholecystitis. Cultures of gallbladder bile however, are only positive in 15% to 30% of cases [15], suggesting that the inflammatory process most often could be of another nature, some suggest inflammation to be caused by a chemical agent [16]. The predominant microorganisms isolated from gallbladder bile in patients with acute cholecystitis are *Escherichia coli *(60%) and *Klebsiella pneumoniae *(22%) [15].

Occlusion of the arterial blood supply to the gallbladder may be a fundamental element in the pathogenesis of acute acalculous cholecystitis [17].

Several groups have tried to induce acute cholecystitis in animal models by combining surgical induced cholestasis with either bacterial infection, chemical irritants and/or gallbladder ischaemia[16,18].

We wanted to induce a severe condition of acute cholecystitis in order to demonstrate the effect of the intervention. Therefore we combined the insults, ligating the cystic duct and artery and inoculation of bacteria.

The aim of the present study was to investigate whether internal drainage of the gallbladder could protect against the development of acute cholecystitis in a pig model.

## Materials and methods

The research protocol was approved by the local research committee (registration number: 2008/561-1489) in accordance with the Danish regulations on animal experiments. Twenty female pigs (Danish Landrace/Yorkshire) with a body weight of approximately 65 kg (Research Centre Foulum under the Danish Institute of Agricultural Sciences) were used for the experiment. Animals were randomized in blocks of four. The pigs were divided into two groups and all of the pigs had acute cholecystitis induced as described below. Drained pigs had an internal double pigtail catheter from the gallbladder to the duodenum. In the undrained pigs the catheter was placed as in drained pigs, but was then immediately removed.

Acute cholecystitis was induced using a combination of previously described models [16,19-22] with slight modifications, thus comprising:

1) Ligation of the cystic artery.

2) Ligation of the cystic duct.

3) Injection of bacteria into the gallbladder lumen.

### Surgery day 0

After premedication with an intramuscular injection of Midazolam 0.4 mg/kg and Ketamine 4 mg/kg, the pigs were intubated and mechanically ventilated (Servo 900 ventilator; Siemens-Elema, Solna, Sweden) with a mixture of air, oxygen and 1.5% isoflurane. Fentanyl was given as a continuous intravenous infusion 10-15 ml/h. A midline laparotomy was performed. The infundibulum of the gallbladder was identified and in its proximity the cystic duct and the cystic artery was dissected and exposed. A duodenotomy was made by an anti-mesenterial incision 1-2 cm distal to the pylorus. The papilla Vateri was identified and cannulated with a catheter (Cook; Angiography catheter HNB7.0-NT-100-M-Ns-CN) which was inserted into the gallbladder and through this a guidewire (Boston Scientific; Jagwire 0.035/450) was placed. After removal of the catheter a double pigtail catheter 7F, 9 cm (Cook; Endoprosthesis T7.0-35-9-35-16S-DENDR) was placed guided by the wire with the proximal tip in the lumen of the gallbladder and the distal tip in the lumen of the duodenum. Hereafter, the guidewire was removed in all animals and in half of them the pigtail catheter was removed. In all pigs, the cystic artery and cystic duct were ligated and the duodenotomy was closed with a single layer continuous seromuscular suture. Finally, 2-4 ml of bile was extracted through the wall of the gallbladder, sent for microbiological examination and replaced with a 2 ml suspension in physiological salt water of an overnight broth culture of bacteria (1 ml of *Escherichia coli *10^5^/ml and 1 ml of *Klebsiella pneumonia *10^5^/ml). The abdomen was closed in two layers with staples in the skin.

### Evaluation day 4

The pigs were killed on day four with a captive bolt pistol. A midline laparotomy was performed and the gallbladder was exposed. Bile was extracted for microbiological analysis and the gallbladder was removed and fixed in the physiological expanded state by injecting 10% formaldehyde solution into the lumen of the gallbladder, thus avoiding artifacts such as changes in wall thickness that can be seen in the collapsed state.

During the experiment, tests were done according to Table 1.

**Table 1:** Overview of test chronology.

Test	Day 0	Day 1	Day 2	Day 3	Day 4
Weight	X				X

Temperature	X	X	X	X	X

Blood samples	X	X	X	X	X

Blood culture					X

Bile culture	X				X

Histology					X

X indicating a test was done

Bloodtests: neutrophils, lymphocytes, eosinophils, monocytes, red blood cells, haemaglobin, haematocrit; liver blood tests: alanin amino transpherase, albumin, alkaline phosphatase, gamma glutamyl transpherase, bilirubin, amylase, acute phase reactants, electrolytes.

Culture of bile and blood: The BacT/ALERT(r) blood culture system (bioMerieux) was used for blood culturing. Ten ml of blood was drawn for an aerobic and an anaerobic standard blood culture bottle and incubated for seven days. Bile aspirates were spread on agar plates and cultured aerobically at 36°C and 5% C0_2 _atmosphere (5% blood agar plate and a MacConkey agar plate, Statens Serum Institut, Copenhagen, Denmark) and anaerobically at 36°C for two days (Anaerobic blood agar plates, Statens Serum Institut, Copenhagen, Denmark). All culture positive isolates were subcultured and identified according to conventional microbiological laboratory methods.

Histological examination: Following fixation, sections of gallbladder extending from the cystic duct to the fundus were embedded in paraffin using standard methods. Paraffin sections were cut at 5 μm and stained with haematoxylin and eosin, and with Masson's trichrome. Histological examination was performed in a blinded manner by an experienced pathologist using a modification of a generally accepted histological scoring system and a model with reproducible histological changes [21].

Each gallbladder in the present study was graded with this scoring system without knowledge of the intervention. The code was subsequently broken and the data was collated.

Briefly, histological score was based on the presence, extent, and severity of haemorrhage, edema, formation of pregranulation tissue (fibroblast proliferation and increased ground substance), lymphatic dilatation, and polymorphonuclear cell (PMN) infiltration in the wall of each gallbladder. Lymphatic dilatation and the presence of PMNs were scored as being either present (1) or absent (0) since their degree of participation in the inflammatory response was essentially all or nothing. Hemorrhage, edema, and pregranulation tissue formation, showed more variation, and were graded accordingly from 0 to 3 (0 = not present, 1 = mild, 2 = moderate, 3 = severe). The individual scores for each histological parameter were added together to give a total possible score of between 0 (histologically normal looking gallbladder, and 11 (the most severely inflamed gallbladder) [21].

### Statistical analysis

Blood, temperature, and weight data from drained and undrained pigs were compared using unpaired parametric (Student's t test) or non-parametric tests (Mann-Whitney U test). Results of blood tests, temperature, and weight were reported as mean and standard error of mean.

The histological scores were compared by category and total score using a non-parametric test (Kruskal-Wallis).

The assumption of normal distribution was evaluated by visual inspection of QQ-plots and histograms. Non-parametric statistics were applied in the case of skewed distribution.

P < 0.05 was considered statistically significant.

## Results

Of the 20 pigs included in the study, 16 survived the full four days after the initial operation leaving eight pigs in each group. Four pigs died, three because of cholascos, one due to gastric retention. There were no significant differences in any blood tests, temperature or weight between the groups.

### Bile

**Figure 1:**
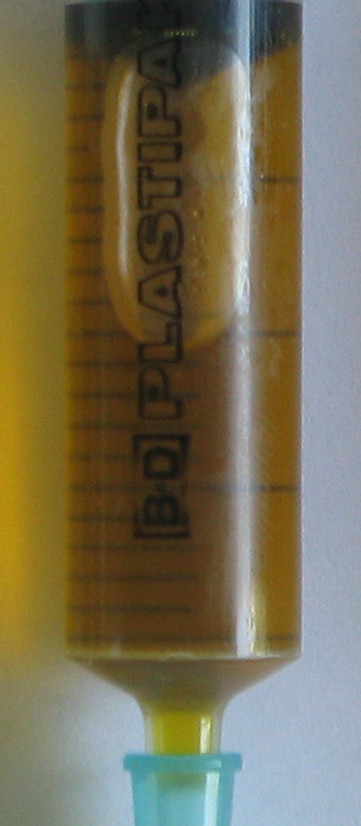
**Bile sample from day 0**.

**Figure 2:**
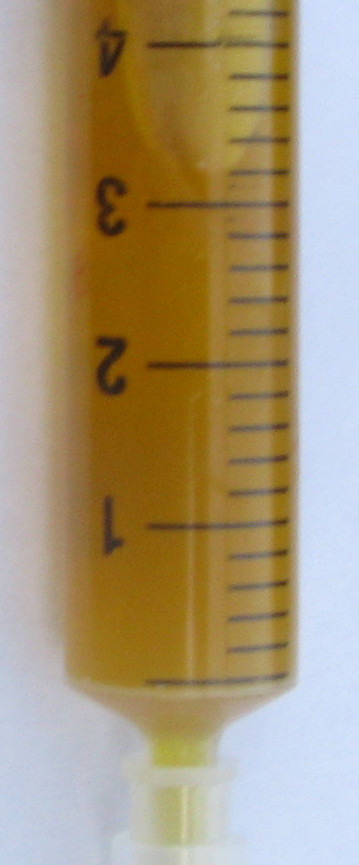
**Bile sample from gallbladder with drainage catheter on day four**.

**Figure 3:**
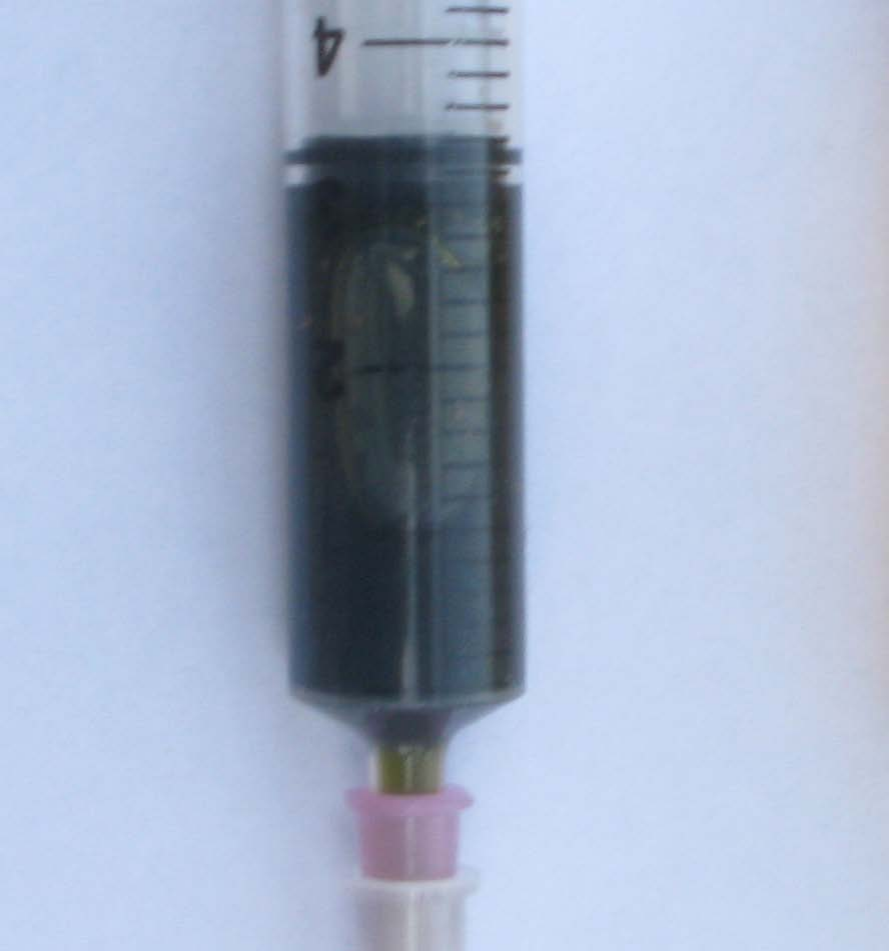
**Bile sample from gallbladder without drainage catheter on day four**.

All drained pigs had a yellowish clear transparent bile on day four, whereas undrained pigs had dark green to black non-transparent bile with sludge (Fig. 1, 2 and 3).

### Bacteria

Nine of sixteen bile samples collected on day 0 in the pigs who survived were non-sterile. *Escherichia coli *alone was found in seven cultures, *Escherichia coli *and *Klebsiella pneumoniae *in one culture and finally *Non-haemolytic Streptococci *in one. On day four a combination of *Escherichia coli *and *Klebsiella pneumoniae *were found in all 14 bile cultures, *Escherichia coli *alone in one and *Klebsiella pneumoniae *alone in one culture. No other bacteria were cultured from the bile samples.

In drained pigs, six and in undrained pigs, eight blood cultures showed bacterial growth on day four; species are shown in Table 2.

**Table 2:** Blood culture results from day four.

Name of bacteria	Drained	Undrained
*Escherichia coli*	5	4

Non-haemolytic *Streptococci*	2	5

*Klebsiella pneumoniae*	3	2

Coagulase-negative *Staphylococci*	1	3

*Enterococci*	2	3

No bacteria	2	0

Eight pigs in each group.

### Histopathology

The inflammation scoring system demonstrated a highly statistically significant difference in all examined parameters. In all instances, scores were higher in gallbladders from undrained pigs (Table 3).

**Table 3:** Histopathological scoring system.

	Lymphatic dilatation (0-1)	Polymorphnuclear cells (0-1)	Hemorrhage (0-3)	Edema (0-3)	Pregranulation tissue (0-3)	Sum (0-11)
Drained	0.25(-0.14; 0.64)	0.25(-0.14; 0.64)	0 (0; 0)	0.25 (-0.14; 0.64)	0.38(-0.06; 0.81)	1.13(-0.39;2.64)

Undrained	1(1;1)	1(1;1)	0.75(0.36;1.14)	1.50 (0.87; 2.13)	2(01.55;2.45)	6.25(5.38;7.12)

p-value	0.0027	0.0027	0.0027	0.0023	0.0008	0.0006

The range of the possible categorical score is indicated in the paragraph in the top line. The scores for drained and undrained are demonstrated as mean (confidence interval). In the bottom line the p-values are presented.

**Figure 4:**
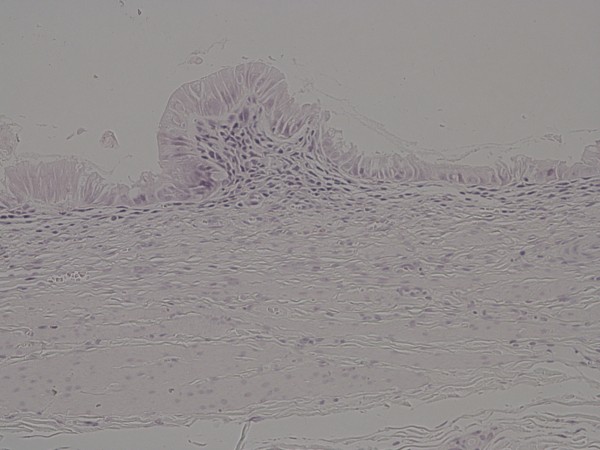
**Gallbladder from drained pig**. The mucosa is intact with a normal number of mononuclear cells (mostly lymphocytes). The muscle and subserosal layers are unremarkable without significant edema, lymphatic dilatation or inflammation. Hematoxylin & eosin.

**Figure 5:**
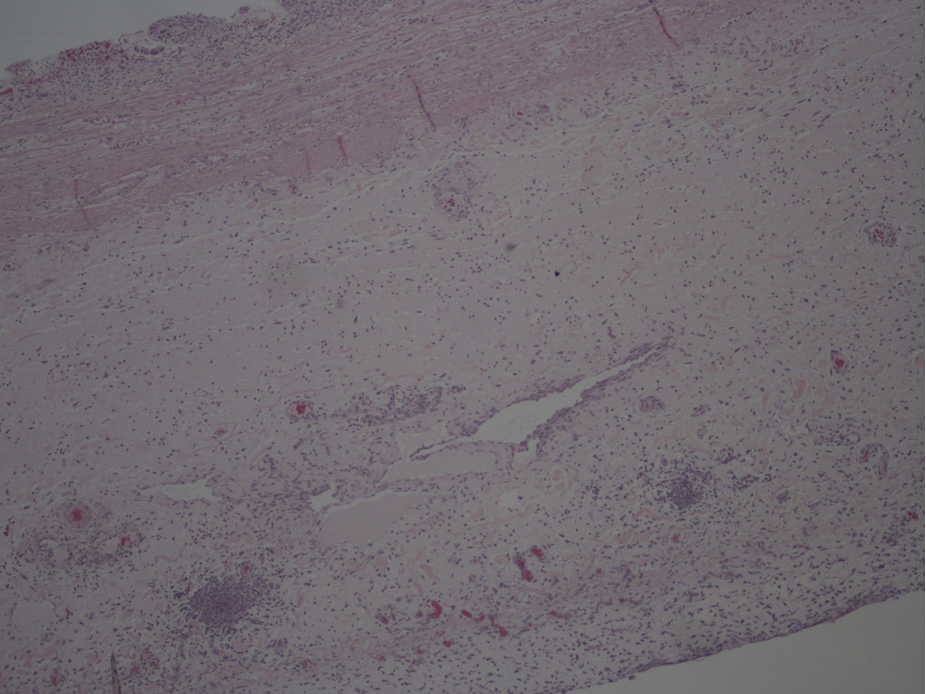
**Gallbladder from undrained pig four days after ligation**. The mucosa, which can just be seen in the upper left-hand part of the figure, shows partial loss. There is inflammation of the underlying muscle layer, whilst the subserosal region is markedly expanded with edema, lymphatic dilatation, formation of pre-granulation tissue and extensive acute inflammation. Hematoxylin & eosin.

**Figure 6:**
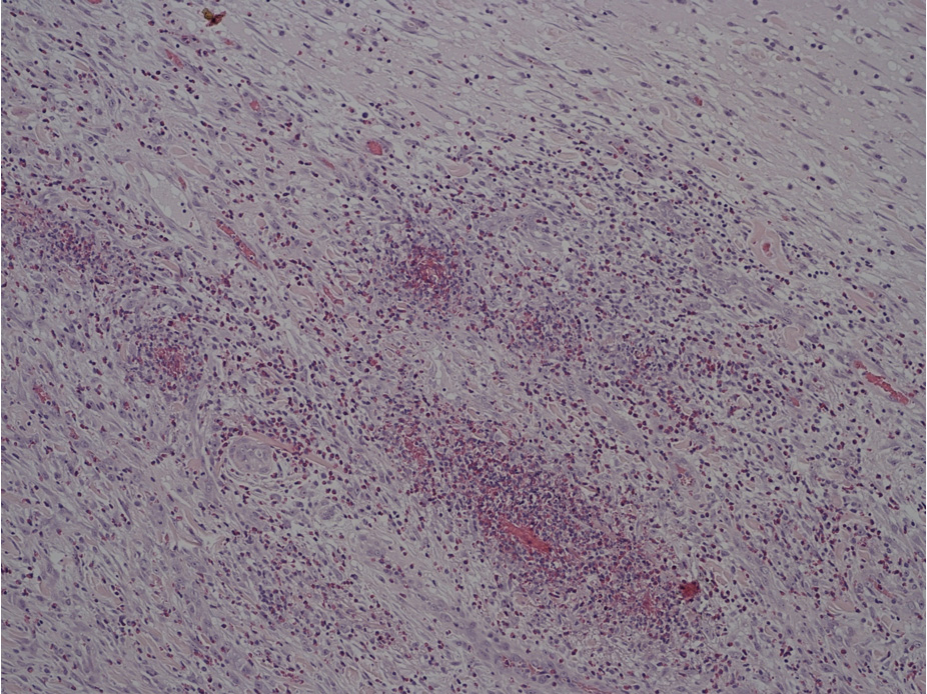
**Higher power picture of Figure 5 of the subserosa showing focal haemorrhage with pre-granulation tissue and marked acute inflammation**. Hematoxylin & eosin.

The histological differences between drained an undrained gallbladders are illustrated in Figure 4, 5 and 6.

## Discussion

The present study in a pig model shows that internal drainage from the gallbladder to the duodenum protects against development of acute cholecystitis judged by a histopathological scoring system. Internal drainage could not prevent the bile from still being infected with bacteria inoculated into the gallbladder lumen four days earlier. There was, however, a distinct difference in the macroscopic appearance in the sense that the bile in the internal drainage group was yellow and transparent whereas it was dark and non-transparent in the undrained pigs.

Multiple factors have been hypothesized to be involved in the development of acute cholecystitis and, therefore, have been used in different animal models. Obstruction of the cystic duct by an impacting gallstone has been the primary causative factor to be proposed, whilst other factors to be implicated include impairment of the gallbladder blood supply, secondary bacterial infection, and abnormal concentrations of bile constituents leading to a chemical inflammation [16,19-22]. In the actual study, acute cholecystitis was induced combining previous described models with modifications comprising ligation of the cystic artery, ligation of the cystic duct and injection of a 2 ml suspension of bacteria (1 ml of *Escherichia coli *10^5^/ml and 1 ml of *Klebsiella pneumonia *10^5^/ml) [16,19-22]. The severeness of the combined insults might explain the high mortality.

Sections from the gallbladders were scored using a previously described histopathological scoring system and showed pronounced signs of inflammation in the group without internal drainage, indicating that the model used for inducing acute cholecystitis in the actual study was adequate [21].

Using a histopathological scoring system based on the presence, extent and severity of hemorrhage, edema, formation of pregranulation tissue, lymphatic dilatation, and PMC infiltration in the gallbladder wall, we found a highly significant protective effect of internal drainage from the gallbladder to the duodenum in the development of acute cholecystitis. These findings support the theory that a gallstone impacting the cystic duct plays a crucial role as a pathogenetic mechanism in the development of acute cholecystitis and it may be an important factor in the maintenance of this disease state. There were only mild degrees of inflammation identifiable microscopically in the internal drainage group, in spite of the fact, that these animals had the cystic artery ligated and had bacteria inoculated into the gallbladder. This suggests that the latter factors are of relatively less importance as single pathogenetic insults compared to outflow impediment in the development of acute cholecystitis. The findings in the actual study also allows us to speculate that internal drainage might be a way to treat already evolved acute cholecystitis.

Surprisingly, bile cultures sampled day 0 showed bacterial growth in nine of 16 cases. This could not be due to contamination from the inoculate, since extraction of bile was done prior to injection of bacteria, but we offer two other explanations:

1. Retrograde contamination from the duodenum while placing the stent, since this was done prior to extraction of bile or

2. In a high proportion of normal pigs, bile is colonized with bacteria from the intestinal tract.

On day four, all bile cultures were contaminated with bacteria in both of the groups, suggesting that infected bile as a sole factor is insufficient to produce acute cholecystitis. These findings also support the above mentioned theory that gallstone impaction in the cystic duct is the key pathogenetical mechanism in the development acute cholecystitis, i.e. acute cholecystitis will not develop if the bile drainage through the cystic duct is preserved in spite of bacterial infection.

Fourteen of 16 pigs showed bacterial growth in blood cultures on day four. Some of the strains cultured (i.e. *Staphylococci *and *Steptococci*) could be due to contamination from the skin. However, *Escherichia coli *and *Klebsiella pneumonia *demonstrated in blood cultures most probably originated from the gallbladder as these were the predominant bacteria cultured from bile. It is not possible to determine whether the positive blood cultures had any effect on the well-being of the animals in the present study as we had no control animals with negative blood cultures.

In conclusion, the present study demonstrated that internal drainage from the gallbladder to the duodenum prevents the development of acute cholecystitis. Since development of acute cholecystitis can be prevented by internal drainage, future studies must demonstrate whether internal drainage can be applicable as a therapeutic intervention in patients suffering from accumulated biliary attacks of pain or in patients already suffering from acute cholecystitis.

## Competing interests

The authors declare that they have no competing interests.

## Authors' contributions

DWK participated in the design of the study, carried out the surgery, drafted the manuscript and performed the statistical analysis. FVM participated in the design of the study and helped to draft the manuscript. JKM was responsible for analysis of cultured bacteria. SJHD did the histological examination and helped to draft the manuscript. PFJ conceived of the study, and participated in its design and coordination and helped to draft the manuscript. All authors read and approved the final manuscript.
